# Evaluation of Physicochemical and Glycaemic Properties of Commercial Plant-Based Milk Substitutes

**DOI:** 10.1007/s11130-016-0583-0

**Published:** 2016-11-05

**Authors:** Stephanie Jeske, Emanuele Zannini, Elke K. Arendt

**Affiliations:** School of Food and Nutritional Sciences, University College Cork, Cork, Ireland

**Keywords:** Plant-based milk substitutes, Protein requirement, Glycaemic index, Dispersion stability

## Abstract

The market for plant-based dairy-type products is growing as consumers replace bovine milk in their diet, for medical reasons or as a lifestyle choice. A screening of 17 different commercial plant-based milk substitutes based on different cereals, nuts and legumes was performed, including the evaluation of physicochemical and glycaemic properties. Half of the analysed samples had low or no protein contents (<0.5 %). Only samples based on soya showed considerable high protein contents, matching the value of cow’s milk (3.7 %). An *in-vitro* method was used to predict the glycaemic index. In general, the glycaemic index values ranged from 47 for bovine milk to 64 (almond-based) and up to 100 for rice-based samples. Most of the plant-based milk substitutes were highly unstable with separation rates up to 54.39 %/h. This study demonstrated that nutritional and physicochemical properties of plant-based milk substitutes are strongly dependent on the plant source, processing and fortification. Most products showed low nutritional qualities. Therefore, consumer awareness is important when plant-based milk substitutes are used as an alternative to cow’s milk in the diet.

**Electronic supplementary material:**

The online version of this article (doi:10.1007/s11130-016-0583-0) contains supplementary material, which is available to authorized users.

## Introduction

Consumer demand for cow’s milk alternatives arose as a result of people being intolerant to cow’s milk, including lactose intolerance and cow’s milk allergy. Nowadays, the avoidance of dairy products is additionally based on health concerns, like cholesterol and antibiotic residues in cow’s milk. Further it is part of different lifestyles including vegetarian and vegan diets or based on ethical considerations against the consumption of cow’s milk. The market for non-dairy milks was growing by 9 % in 2015 to reach $1.9 billion [1] with 138 different variants of plant-based milk substitutes (PBMS’s) just in Europe [2]. Generally, PBMS’s are extracts of plant material in water, which resemble cow’s milk in appearance. Technologically, water is used to extract the plant material, followed by the liquid separation for the beverage production. Processing steps beforehand and afterwards like the addition of other ingredients, heat treatments or homogenisation can be applied to formulate the final product [3]. Soya milk is the most common milk substitute. However, 14 % of the individuals who suffer from cow’s milk allergy also have reactions against soya [4]. Beside soya, other plant sources are used for developing non-dairy milk products, like oat, almond, coconut, rice and quinoa and their market share is increasing. Consumers are purchasing PBMS’s for their health and wellness benefits [1, 5]. Hence, health claims regarding vitamins, fibre, or cholesterol values are very common in this category. Sixty-nine % of Americans trust that non-dairy milks are nutritious for kids [1]. Nevertheless, some products have extremely low protein contents. Recently, Mäkinen et al. [3] pointed out the risk of replacing cow’s milk with PBMS’s, especially for young children, and emphasised the importance of consumer awareness. However, the choice of raw material has a great impact on the product quality as well as the process technology adopted. Due to the fact that soya-based milk substitutes (BMS’s) are already successful on the market since more than 70 years, a lot of research and literature is available. A huge selection of different soya beans with special designed processing characteristics or physical properties are available [6]. On the other hand there are more and more varieties of PBMS’s available on the market but the quality of those products has not been yet fully investigated. Therefore, a screening of 17 different commercial milk substitutes, based on different cereals, nuts and legumes was performed. The aim of this study is to give an overview on physicochemical and nutritional properties of different PBMS’s.

## Materials and Methods

### Samples

Seventeen different commercial PBMS’s based on different cereals, nuts and legumes were purchased on the market (supplementary Table 1 shows the list of samples selected for this research). Two different batches of each commercial sample were analysed. Bovine milk was used as a control. The samples were stored at 4 °C and used within 2–3 days of opening the packaging.

### Composition

Total nitrogen content of samples was analysed using Kjeldahl method (MEBAK 1.5.2.1). Nitrogen to protein conversion factors were selected according to WHO/FAO [7] and plant ingredient; 5.18 for nut- and seed-BMS’s, 5.83 for oat-, 5.95 for rice-, 5.71 for soya-BMS’s and 6.38 for bovine milk. Moisture was determined by drying in an oven at 103 °C until constant mass was reached. Ash was analysed by incineration in a muffle furnace: samples were pre-heated in crucibles for 1 h at 100 °C and ashed for 4 h at 600 °C. Fat content was determined by extracting total fat using Soxhlet technique with hot solvent and gravimetry. For sugar analysis, samples were filtered (0.25 μm) and diluted with water. Sugar profiles were analysed using an Infinity 1260 HPLC system equipped with a refractive index detector (Agilent Technologies, Palo Alto, CA) with a Hi-Plex H, 300 × 7.7 mm, 8 μm HPLC column (Agilent Technologies, Palo Alto, CA) at a flow rate of 1 mL/min of water. Sugar concentrations were determined using sucrose, maltose, glucose, fructose, lactose and galactose as external standards. For maltose and sucrose qualification samples were diluted with water/acetonitrile (75:25 *v/v*) and analysed with a Supelcosil LC-NH_2_, 250 × 4.6 mm, 5 μm HPLC column (Sigma-Aldrich) at a flow rate of 1 mL/min of water/acetonitrile (75:25 *v/v*) using the same HPLC system. Sum of sugars was determined as the sum of all sugars detected in one sample. Total starch was determined using the enzyme kit K-TSTA supplied by Megazyme, Ireland.

### Glycaemic Index and Glycaemic Load

*In vitro* determination of the glycaemic index (GI) was evaluated according to Magaletta & DiCataldo [8] using a calculation designed by an artificial neural network. An equivalent to 0.5 g of available carbohydrates (based on the results of sugar and starch analysis) was digested by a multi-enzyme preparation. The digestate was analysed for glucose, fructose, lactose and galactose with the HPLC described as above. These results, together with the results from the protein and fat content of the samples, were used to feed the calculation:

1 $$ \begin{array}{l}GI= 26.264529- 1.048186\cdot Protein\ \left[\%\right]- 0.248138\cdot Fat\ \left[\%\right]\\ {}+ 621.7824\cdot Glucose\ \left[\%\right]- 52.7993\cdot Fructose\ \left[\%\right]\\ {}- 233.67679\cdot Lactose\ \left[\%\right]- 61.21071\cdot Galactose\ \left[\%\right]\end{array} $$

Glycaemic load (GL) was calculated according to Atkinson et al. [9]:

2 $$ GL = \left(GI\cdot available\  carbohydrate\ (g)\  per\  portion\right)/ 100 $$

The portion size was set to 250 g.

### Colour Measurement

The colour values were measured using the CIE L*a*b* colour system and obtained using illuminant D65. The instrument used was a colorimeter (CR-400, Konica Minolta, Osaka, Japan). Colour of samples was characterised according to whiteness index (WI), defined as:

3 $$ WI=100-\sqrt{\left.{\left(100-{L}^{*}\right)}^2+{a}^{*2}+{b}^{*2}\right)} $$

### Physicochemical Properties

#### Rheology

The rheological behaviour of the products was characterised using a controlled stress rheometer (MCR301, Anton Paar GmbH, Austria) equipped with a sensor system of coaxial cylinders (C-CC27-T200/SS, Anton Paar GmbH, Austria). The shear stress (σ) was measured as a function of shear rate ($\overset{\cdot }{\mathrm{y}}$) ranging from 0.5 to 100 s^−1^ within 500 s. The power law model was fitted to the experimental points to determine the flow behaviour index (n):

4 $$ \sigma =K{\left(\overset{\cdot }{y}\right)}^{n-1} $$

The measurements were carried out at 10 °C. The apparent viscosity measured at 10 s^−1^ is referred to as “viscosity”.

#### Stability

Stability was determined through phase separation analysis using an analytical centrifuge (LUMiSizer; LUM GmbH, Berlin, Germany). The instrumental parameters used were as follow: 1000 rpm for 30 min followed by 3000 rpm for 60 min at 24 °C. Height of sediment and creaming layer in mm, and separation rate in %/h were determined.

#### Particle Size Distribution

Analyses of particle size distribution were carried out using a static laser light diffraction unit (Mastersizer 3000, Malvern Instruments Ltd, Worcestershire, UK). Samples were applied to the instrument with distilled water as dispersion medium at 2,800 rpm until an obscuration rate of 5 % was obtained. The refractive index was determined using a hand-held refractometer (Atago R5000, Atago, Tokyo, Japan).

### Statistical Analysis

All analyses were carried out at least in triplicate. Means were compared using one-way analysis of variance (ANOVA) and Tukey’s *post hoc*-test using Minitab release 16 (Minitab Inc. State College, Pa., USA). The level of significance was determined at *p* < 0.05. Linear correlation measurements of results were performed using Pearson’s correlation.

## Results and Discussion

### Composition and Glycaemic Response

The compositional data of the samples is given in Tables 1 and 2. The protein content ranged from 0.07 % for brown rice-BMS to 3.70 % for bovine milk and the soya-BMS from Sojade. Half of the analysed samples had low or no protein contents (<0.5 %). Only samples based on soya showed considerable high protein contents. Compared to the labelled values most of the measured values coincide but two, which differed greatly. The measured values of the quinoa- and Provamel soya-based samples were 85 and 26 % lower than the labelled values. However, this is still within the discrepancy range, set by the European Commission [10]. In addition to the low protein content in most of the PBMS’s, plant proteins have a lower protein quality in terms of digestibility compared to animal derived proteins; cow’s milk protein has a protein digestibility-corrected amino acid score of 121, whereas literature values for the plant proteins are all lower: soya, cashew, quinoa, rice, hemp, oat and almond have values of 91, 90, 67, 56, 49–53, 41–51, and 23, respectively [11–17]. Hence, if these products are consumed to replace cow’s milk in the diet and used as a protein source, this can cause a protein deficit and severe illnesses. Especially for young infants, an appropriate healthy diet tailored to their requirements is important since about 40 % of the protein is needed for growth. Adults need protein for maintenance, therefore their protein requirements in g/kg/d is lower. Currently, the protein requirement stated by the recent FAO report is 0.66 g of protein /kg/d for adults and 1.12 g of protein /kg/d for 0.5 year old infants [18]. Le Louer et al. [19] reported severe malnutrition of young infants with inappropriate PBMS consumption. The children suffered from various diseases, like protein-calorie malnutrition and deficiencies of minerals and vitamins, with severe consequences. Fat content, shown in Table 1, was high for bovine milk at 3.28 %. Almond-based samples from The good little Cook and Provamel as well as the carob/almond-BMS exceeded this level, while samples based on coconut, oat, rice and brown rice contained <1 % fat. Besides the quantity of fat the quality is of interest; bovine milk is high in saturated fatty acids with about 2.5 g/100 g, whereas plants have generally a low content [20]. Furthermore, the hemp-based sample claims to be a source of Omega-3 & 6. The soya based product from alpro contained the highest ash content at 0.99 %, as it contains added calcium. The other samples had lower values than bovine milk with 0.62 % (Table 1). Bovine milk is an important food source for several minerals *e.g*., calcium, potassium, magnesium and iodine [21]. PBMS’s are often fortified with minerals to prevent deficiencies compared to cow’s milk; seven of the samples contain calcium salts. On the other hand, pollution, like high arsenic levels in rice is a well-known problem, A study by Meharg et al. [22] revealed that 19 out of 19 rice-BMS’s exceeded the inorganic arsenic EU and US limits for drinking water standards. The starch content for all the PBMS’s was low and did not significantly differ among the samples evaluated (Table 1) with the exception of cashew-BMS and the cereal-BMS’s, including oat, rice and brown rice where the starch detected was at 0.73, 2.00, 0.54 and 1.17 %, respectively. Some of the PBMS’s contain added sugars or sweeteners, which contributed to their main sugar and resulted in comparatively high total sugar contents. Samples based on cashew, macadamia, and quinoa were sweetened with agave syrup, the organic soya drink from Provamel contained apple concentrate. Both sweeteners are high in fructose, resulting in high fructose levels for those PBMS’s (<1.27 %). Samples high in sucrose were sweetened with sucrose (Almond original, Hazelnut original, Soya original from alpro) or maple syrup (carob/almond-BMS) with high values above 2.88 %. Another source of sugar is the starch hydrolysis step during processing. Products containing ingredients high in starch are naturally high in maltose and, or glucose. The oat-BMS contained high amounts of maltose at 3.34 %, but the rice-PBM’s were high in both, maltose and glucose, and resulted in total amounts of sugar at 7.02 and 5.58 % respectively. Bovine milk contained 3.33 % lactose and 0.05 % galactose only, whereas none of the BPMS’s contained lactose or galactose. Carbohydrates are digested and absorbed in the blood as glucose to provide energy. The blood glucose level affects the human metabolism greatly and is strictly controlled by peptide hormones like glucagon and insulin [23]. A way to qualify the effect of food on the blood sugar level is the GI; it represents the post-prandial uptake of glucose into the blood compared to a reference [24]. The type of carbohydrate is the main factor accounting for the glycaemic response since all the carbohydrates follow different metabolic pathways to be transformed to glucose and enter the blood [23]. The glucose concentration correlated with the *in vitro* GI (0.80, *p* < 0.001). Hence, samples containing mainly glucose such as coconut- and rice-BMS’s had a high GI (>96). The samples contained as well maltose, sucrose, fructose and lactose. These sugars have a GI by itself of 105, 61, 19 and 46 respectively [25]. Generally, the sugar type governs the value of GI. Just the oat-based PMS was an exception in this study. Even though it contained mainly maltose it resulted in a moderate GI of 59. This can be explained by the β-glucan content in oats, which is known to reduce the GI [26]. The GI can be classified into three categories: values ≤55 are defined as low, 56–69 as medium, foods having a GI ≥70 are defined as high [9]. Bovine milk and 8 samples including the products based on almond from Provamel, carob/almond, cashew, macadamia, quinoa and soya (from Provamel, Sojade and alpro (wholegrain)) had low GI values. Five samples had medium GI values and both of the rice based products as well as the coconut-BMS resulted in a high GI greater than 97 (Table 2). Literature values are rare for PBMS’s, but values for soya-, rice- and quinoa-BMS’s were found [9, 25, 27] and are in accordance with the values in this work. As recommended by the American Diabetes Association [28] not the source or type of carbohydrates is decisive for a healthy diet, but the total amount, which effects the GI in foods. A good tool to measure this is the GL, indicating the effect of one food serving on the blood glucose level after consumption. The rice-BMS’s, which had a high GI showed as well a high GL value (18.33 and 16.85) since they contained a lot of carbohydrates. These values are comparable to Coca-Cola or cakes [25]. However, considering the GL value, the rest of the samples showed low to moderate values. Bell & Sears [24] reviewed the impact of GL on the human health. They came to the conclusion that a low GL diet reduces the risk for e.g. cardiovascular disease, obesity and diabetes. Therefore, attention should be brought to this value and some of these milk substitutes cannot be perceived as healthy, but should be handled as a treat. Additionally, bovine milk is an important food source for vitamin A, D, B12 and riboflavin [21]. These results and other research showed, avoiding dairy products is resulting in a nutrient deficiency and does generally not result in a nutritionally equivalent diet [29, 30].

**Table 1:** Composition in g/100 g and whiteness index [-] of plant-based milk substitutes and bovine milk

Brand	Name	Protein	Fat	Ash	Starch	Whiteness index
The good little Cook	Almond MLK	2.11 ± 0.09^d^	4.40 ± 0.11^a^	0.35 ± 0.04^fg^	0.06 ± 0.00^g^	68.36 ± 1.58^efgh^
alpro	Almond original	0.41 ± 0.02^fg^	1.18 ± 0.05^fgh^	0.55 ± 0.00^cd^	0.06 ± 0.00^g^	72.57 ± 0.09^cd^
Provamel	Organic almond drink	0.95 ± 0.39^e^	3.69 ± 0.11^ab^	0.21 ± 0.02^ij^	0.07 ± 0.01^fg^	75.95 ± 0.66^b^
The good little Cook	Carob almond MLK	2.4 ± 0.24^cd^	3.35 ± 1.73^abc^	0.36 ± 0.00^ef^	0.07 ± 0.01^g^	51.57 ± 0.18^l^
Provamel	Organic cashew drink	0.87 ± 0.10^e^	2.50 ± 0.10^cde^	0.23 ± 0.01^hij^	0.73 ± 0.07^c^	65.57 ± 0.52^hi^
alpro	Coconut original	0.08 ± 0.00^g^	0.84 ± 0.00^gh^	0.52 ± 0.01^d^	0.13 ± 0.02^fg^	67.75 ± 2.70^fgh^
alpro	Hazelnut original	0.36 ± 0.00^fg^	1.56 ± 0.05^defg^	0.52 ± 0.00^d^	0.04 ± 0.00^g^	56.31 ± 0.07^k^
Braham & Murray	Hemp milk unsweetened	0.08 ± 0.04^g^	2.44 ± 0.23^cde^	0.42 ± 0.01^e^	0.05 ± 0.01^g^	68.49 ± 0.02^efgh^
Provamel	Organic macadamia drink	0.29 ± 0.01^g^	2.62 ± 0.06^bcd^	0.25 ± 0.01^hi^	0.21 ± 0.11^efg^	51.73 ± 1.18^l^
Oatly	Organic oat drink	0.70 ± 0.19^ef^	0.38 ± 0.06^h^	0.2 ± 0.00^ijk^	2.00 ± 0.20^a^	60.21 ± 4.46^j^
EcoMil	Quinoa drink	0.22 ± 0.04^g^	2.32 ± 0.13^cde^	0.17 ± 0.01^jk^	0.19 ± 0.05^efg^	71.35 ± 0.20^cde^
Vitariz	Organic rice drink natural	0.32 ± 0.04^fg^	0.85 ± 0.06^gh^	0.14 ± 0.02^kl^	0.54 ± 0.04^d^	66.49 ± 3.94^ghi^
Rude Health	Organic brown rice drink	0.07 ± 0.00^g^	0.95 ± 0.04^gh^	0.10 ± 0.010^l^	1.17 ± 0.08^b^	63.47 ± 3.91^i^
Provamel	Organic soya drink, calcium	2.72 ± 0.06^c^	2.11 ± 0.04^def^	0.61 ± 0.04^bc^	0.10 ± 0.07^fg^	70.34 ± 0.04^def^
Sojade	Plain UHT organic soya drink	3.70 ± 0.03^a^	2.04 ± 0.11^def^	0.34 ± 0.02^fg^	0.11 ± 0.03^fg^	74.49 ± 0.01^bc^
alpro	Soya organic, wholebean	3.16 ± 0.32^b^	1.77 ± 0.06^defg^	0.29 ± 0.00^gh^	0.10 ± 0.01^fg^	69.27 ± 0.69^efg^
alpro	Soya original	2.61 ± 0.13^c^	1.48 ± 0.05^efg^	0.99 ± 0.08^a^	0.08 ± 0.01^fg^	74.56 ± 0.10^bc^
Clona Dairy Product Ltd.	Fresh milk, pasteurised and homogenised	3.70 ± 0.14^a^	3.28 ± 0.05^bc^	0.62 ± 0.01^b^	0.06 ± 0.01^g^	81.89 ± 0.01^a^

Values within a column that share a superscript are not significantly different from one another (*p* < 0.05)

**Table 2:** Sugar compositions (except lactose and galactose, stated in the text) in g/100 g, and glycaemic properties [-] of plant-based milk substitutes and bovine milk

Brand	Name	Glucose	Fructose	Sucrose	Maltose	Sum of sugars	Glycaemic index	Glycaemic load
The good little Cook	Almond MLK	0.06 ± 0.00^e^	n.d.	0.52 ± 0.02^d^	n.d.	0.58 ± 0.02^i^	58.68 ± 3.61^bcde^	0.94 ± 0.05^g^
alpro	Almond original	0.22 ± 0.00^de3^	0.06 ± 0.01^fg^	3.42 ± 0.03^a^	n.d.	3.69 ± 0.05^de^	49.10 ± 2.53^ef^	4.60 ± 0.30^e^
Provamel	Organic almond drink	n.d.	n.d.	0.16 ± 0.01^gh^	n.d.	0.16 ± 0.01^i^	64.21 ± 1.96^b^	0.37 ± 0.00^g^
The good little Cook	Carob almond MLK	0.87 ± 0.02^c^	0.61 ± 0.00^e^	3.10 ± 0.14^b^	n.d.	4.58 ± 0.16^c^	54.33 ± 1.10^bcdef^	6.32 ± 0.25^d^
Provamel	Organic cashew drink	0.49 ± 0.03^cde^	1.96 ± 0.06^c^	0.43 ± 0.01^de^	n.d.	2.87 ± 0.09^fg^	52.82 ± 3.77^cdef^	4.76 ± 0.28^e^
alpro	Coconut original	0.81 ± 0.00^cd^	n.d.	n.d.	1.05 ± 0.11^d^	1.86 ± 0.11^h^	96.82 ± 5.05^a^	4.81 ± 0.01^e^
alpro	Hazelnut original	n.d.	n.d.	3.09 ± 0.01^b^	n.d.	3.09 ± 0.01^efg^	55.76 ± 0.24^bcdef^	4.37 ± 0.02^e^
Braham & Murray	Hemp milk unsweetened	0.02 ± 0.03^e^	0.01 ± 0.02^g^	0.05 ± 0.01^h^	n.d.	0.09 ± 0.04^i^	59.94 ± 1.28^bc^	0.21 ± 0.04^g^
Provamel	Organic macadamia drink	0.30 ± 0.03^cde^	2.23 ± 0.11^ab^	0.26 ± 0.03^fg^	n.d.	2.79 ± 0.12^fg^	49.47 ± 0.57^def^	3.71 ± 0.18^ef^
Oatly	Organic oat drink	0.01 ± 0.00^e^	n.d.	n.d.	3.34 ± 0.17^a^	3.35 ± 0.18^ef^	59.61 ± 5.44^bcd^	7.98 ± 0.71^c^
EcoMil	Quinoa drink	0.43 ± 0.03^cde^	2.34 ± 0.13^a^	n.d.	0.43 ± 0.11^e^	3.2 ± 0.27^efg^	53.28 ± 0.70^cdef^	4.51 ± 0.24^e^
Vitariz	Organic rice drink natural	4.12 ± 0.79^a^	0.07 ± 0.01^fg^	n.d.	2.83 ± 0.29^b^	7.02 ± 1.09^a^	97.74 ± 6.81^a^	18.33 ± 1.28^a^
Rude Health	Organic brown rice drink	3.07 ± 0.06^b^	0.10 ± 0.02^fg^	n.d.	2.41 ± 0.02^c^	5.58 ± 0.03^b^	99.96 ± 5.75^a^	16.85 ± 1.02^b^
Provamel	Organic soya drink, calcium	0.50 ± 0.08^cde^	1.27 ± 0.02^d^	n.d.	0.66 ± 0.02^e^	2.43 ± 0.05^efg^	47.53 ± 4.07^f^	3.01 ± 0.23^f^
Sojade	Plain UHT organic soya drink	0.52 ± 0.58^cde^	n.d.	0.36 ± 0.01^ef^	n.d.	0.88 ± 0.57^i^	54.02 ± 8.76^bcdef^	1.24 ± 0.51^g^
alpro	Soya organic, wholebean	0.01 ± 0.00^e^	n.d.	0.35 ± 0.02^ef^	n.d.	0.36 ± 0.02^i^	49.49 ± 2.75^def^	0.57 ± 0.01^g^
alpro	Soya original	0.15 ± 0.05^e^	0.06 ± 0.02^fg^	2.88 ± 0.07^c^	n.d.	3.09 ± 0.11^efg^	61.50 ± 3.75^bc^	4.87 ± 0.14^e^
Clona Dairy Product Ltd.	Fresh milk, pasteurised & homogenised	n.d.	n.d.	n.d.	n.d.	3.38 ± 0.04^efa^	46.93 ± 0.53^f^	4.03 ± 0.02^e^

Values within a column that share a superscript are not significantly different from one another (*p* < 0.05)

*n.d*. refers to not detectable

^a^Cow’s milk consisted of 3.33 % lactose and 0.05 % galactose, none of the PBMS’s contained lactose or galactose

### Whiteness Index

The WI is given in Table 1. The colour of food is one of the first properties observed by consumers, influencing choice and preference. The WI is one of the most important quality parameters for milk [31]. The WI for bovine milk is the highest with 81.89 and all PBMS’s appeared darker (*p* < 0.05). Raw material and processing steps are influencing WI; however all samples appeared more or less dark and brown to yellow. The WI ranged from 52 for carob/almond- and macadamia-BMS’s up to 75 for soya (-original, alpro)-BMS, which made all of the samples easily distinguishable from cow’s milk.

### Physicochemical Properties

The physicochemical properties are summarised in Table 3. Bovine milk is a natural emulsified beverage; indeed it showed the best performance throughout all of the tests. The apparent viscosity was low and no significant differences could be found to bovine milk with a viscosity of 3.15 mPa∙s for most of the samples. Products based on coconut and almond from Provamel had a viscosity of 47.80 and 26.23 mPa∙s and showed a pseudoplastic behaviour with a flow index of 0.40 and 0.56. Almond (alpro)-, quinoa-, hazelnut-, hemp- and soya (-original, alpro)-based samples showed as well higher viscosities than bovine milk and pseudoplastic behaviour. All of them contained hydrocolloids like locust bean gum, carrageenan or xanthan gum (see supplementary Table 1). Hydrocolloids increase the viscosity and have an impact on the flow behaviour [32]. Only the soya- (original, alpro) based sample had a considerably low viscosity and newton like flow behaviour, even though it contained gellan gum. Considering the particle size measurements, volume mean diameters (d_4.3_) showed no significant differences for all samples with low values varying from 0.60 to 10.51 μm, but quinoa- and cashew-BMS’s, with values of 81.47 and 29.17 μm respectively. Considering the mean diameters, it should be noted that the d_4,3_ is sensitive to the presence of larger particles, and the d_3,2_ parameter to smaller particles. Evaluating the d_3,2_ mean diameter for the quinoa- and cashew-BMS’s, it is evident that just a small amount of big particles was present in these samples, since they did not have the highest values. PBMS’s are produced by disintegration of plant materials, which means that particle composition and size is not as uniform as in bovine milk, which had significantly the lowest d_3,2_ and d_4,3_ values (0.36 and 0.60 μm). The samples which were similar to bovine milk, with low d_4.3_ (≤3.43 μm) values showed a monomodal particle size distribution. Only almond- (Provamel), cashew-, oat-, quinoa- and rice-BMS’s showed a polydisperse distribution. Chu et al. [33] found that polydispersity of particles in colloidal dispersions leads to destabilisation of the system. Indeed, it was found that the polydisperse samples had generally high separation rates (>27 %/h). Numerical data describing the stability of the beverages are presented in Table 3. Bovine milk was the most stable one with a separation rate of 3.87 %/h. Samples based on almond (alpro), macadamia, hemp, hazelnut and soya (Sojade and Provamel) showed considerable stabilities with values <10 %/h. Whereas the rest of the samples were unstable and some separated rapidly including almond (The good little Cook)-, carob/almond-, macadamia- and brown rice-BMS’s with values >50 %/h. The markedness and velocity of sedimentation or creaming depends also on the viscosity of the continuous phase and the density difference between particles and continuous phase [34]. The viscosity was high for samples containing stabilisers. Stabilisers improve the stability by simply increasing the viscosity of the continuous phase. Most of the samples (almond- (alpro), hazelnut- and hemp-BMS’s) containing this kind of additives indeed showed a good stability. Denser particles sediment while the lighter ones cream on top of the liquid. Only the oat-BMS had a considerable creaming layer (2.48 mm). This sample showed as well a thick sedimentation layer (3.51 mm). Together with samples based on carob/almond and cashew, it was the only sample, which had a significantly thicker sediment layer than bovine milk.

**Table 3:** Physicochemical properties of plant-based milk substitutes and bovine milk

Brand	Name	Separation rate [%/h]	Sediment [mm]	Creaming [mm]	Viscosity [mPa∙s]	Flow index [-]	d_3,2_ [μm]	d_4,3_ [μm]
The good little Cook	Almond MLK	52.42 ± 2.48^a^	0.00 ± 0.00^c^	0.68 ± 0.21^bc^	4.63 ± 0.98^gh^	0.82 ± 0.11^cde^	2.36 ± 0.17^a^	0.90 ± 0.02^c^
alpro	Almond original	1.35 ± 0.55^k^	0.00 ± 0.00^c^	0.00 ± 0.00 ^c^	19.08 ± 1.98^cf^	0.70 ± 0.04^efg^	1.10 ± 0.01^fg^	1.84 ± 0.11^c^
Provamel	Organic almond drink	30.17 ± 4.97^fgh^	1.72 ± 0.76^abc^	1.16 ± 0.60^b^	26.32 ± 7.03^b^	0.56 ± 0.21^g^	2.09 ± 0.27^b^	5.96 ± 1.84^c^
The good little Cook	Carob almond MLK	51.7 ± 1.83^a^	3.93 ± 1.79^a^	0.81 ± 0.28^bc^	3.87 ± 0.85^h^	0.98 ± 0.04^ab^	1.48 ± 0.04^de^	2.59 ± 0.20^c^
Provamel	Organic cashew drink	27.46 ± 8.31^gh^	4.22 ± 2.91^a^	0.98 ± 0.18^bc^	5.57 ± 0.80^gh^	0.97 ± 0.03^ab^	2.30 ± 0.49^a^	29.17 ± 24.65^b^
alpro	Coconut original	37.43 ± 1.06^def^	2.60 ± 0.31^abc^	0.60 ± 0.01^bc^	47.80 ± 2.16^a^	0.40 ± 0.03^h^	1.34 ± 0.01^e^	1.72 ± 0.13^c^
alpro	Hazelnut original	1.27 ± 0.26^k^	0.00 ± 0.00^c^	0.00 ± 0.00 ^c^	24.80 ± 1.45^bcd^	0.67 ± 0.04^fg^	1.52 ± 0.07^d^	2.21 ± 0.11^c^
Braham & Murray	Hemp Milk Unsweetened	4.44 ± 3.98^jk^	0.26 ± 0.30^c^	0.00 ± 0.00^c^	25.00 ± 7.27^bc^	0.73 ± 0.05^ef^	1.06 ± 0.16^fg^	1.51 ± 0.09^c^
Provamel	Organic macadamia drink	54.39 ± 3.91^a^	0.29 ± 0.07^c^	0.97 ± 0.03^bc^	2.22 ± 0.31^h^	1.01 ± 0.03^a^	1.77 ± 0.02^c^	3.43 ± 0.08^c^
Oatly	Organic oat drink	40.13 ± 3.13^cde^	3.51 ± 0.60^ab^	2.48 ± 1.05^a^	6.77 ± 1.03^gh^	0.89 ± 0.02^abcd^	1.7 ± 0.07^c^	3.83 ± 0.53^c^
EcoMil	Quinoa drink	32.01 ± 5.55^efg^	1.08 ± 0.10^bc^	0.64 ± 0.19^bc^	13.20 ± 2.20^ef^	0.76 ± 0.07^def^	1.12 ± 0.02^f^	81.47 ± 39.81^a^
Vitariz	Organic rice drink natural	42.83 ± 1.23^bcd^	0.36 ± 0.23^c^	0.74 ± 0.16^bc^	2.77 ± 0.06^h^	0.97 ± 0.03^ab^	0.88 ± 0.09^hi^	10.51 ± 13.20^c^
Rude Health	Organic brown rice drink	50.86 ± 0.44^ab^	2.15 ± 0.02^abc^	0.64 ± 0.06^bc^	2.21 ± 0.02^h^	1.02 ± 0.01^a^	0.63 ± 0.00^j^	0.72 ± 0.05^c^
Provamel	Organic soya drink, calcium	11.33 ± 3.22^ij^	2.65 ± 2.40^abc^	0.65 ± 0.76^bc^	7.58 ± 4.84^fgh^	0.91 ± 0.10^abc^	0.94 ± 0.07^ghi^	1.28 ± 0.13^c^
Sojade	Plain UHT organic soya drink	8.61 ± 1.41^ijk^	1.15 ± 0.25^bc^	0.00 ± 0.00^c^	3.49 ± 0.10^h^	1.01 ± 0.02^a^	0.80 ± 0.00^i^	0.99 ± 0.01^c^
alpro	Soya organic, wholebean	13.27 ± 0.78^i^	0.20 ± 0.19^c^	0.00 ± 0.00^c^	2.57 ± 0.04^h^	1.00 ± 0.02^a^	0.85 ± 0.02^i^	1.01 ± 0.06^c^
alpro	Soya original	22.56 ± 3.88^h^	0.60 ± 0.37^c^	0.91 ± 0.45^bc^	5.98 ± 0.22^gh^	0.92 ± 0.01^abc^	0.94 ± 0.00^ghi^	1.22 ± 0.01^c^
Clona Dairy Product Ltd.	Fresh milk, pasteurised and homogenised	3.87 ± 0.17^jk^	0.60 ± 0.05^c^	0.70 ± 0.27^bc^	3.15 ± 0.01^h^	1.01 ± 0.02^a^	0.36 ± 0.03^k^	0.60 ± 0.02^c^

Values within a column that share a superscript are not significantly different from one another (*p* < 0.05)

## Conclusion

This study showed that PBMS’s differ remarkably in nutritional and physicochemical properties. Depending on the raw material, some had very low protein contents and high glycaemic values. If these products are portrayed as cow’s milk substitutes, the nutritional inferiority can cause severe illnesses. To the authors’ knowledge, this paper presents the first assessment of many PBMS’s, taking several nutritional values into account. Especially the determination of GI values gave new insights to evaluate the nutritional importance. Moreover, stability and rheology properties were poor and only products based on soya showed good performances without containing hydrocolloids. PBMS’s have a reputation to be healthy and nutritionally valid [1, 5], but this study unveiled that most products lack in nutritional quality. Only soya based substitutes showed overall good results, comparable to cow’s milk. Manufactures need to improve these, *e.g*., by choosing adequate raw materials as well as tailored and consumer-friendly processing technologies (*i.e*., the application of enzymes and/or fermentation technology), rather than adding low-cost fortifiers and additives like sweeteners and gums. More research is needed in this field to gain knowledge and to overcome issues regarding nutrition and stability. Further, the development of new milk alternatives that cause no adverse effects in humans and that have better nutritional, sensory and technological properties is necessary.

**Abbreviations**

BMS Based milk substitutes
GI Glycaemic index
GL Glycaemic load
PBMS Plant-based milk substitutes
WI Whiteness index

## Electronic supplementary material

Below is the link to the electronic supplementary material.

ESM 1 (DOCX 38 kb)
