# Supplementary material for: Evaluation of Physicochemical and Glycaemic Properties of Commercial Plant-Based Milk Substitutes
Source: Plant Foods Hum Nutr. 2016 Nov 5;72(1):26–33. doi: 10.1007/s11130-016-0583-0 (PMC5325842; doi:10.1007/s11130-016-0583-0)
Supplement: Supplementary file 1 — (DOCX 38 kb) [file 11130_2016_583_MOESM1_ESM.docx]

Table 1 List of selected commercial plant-based milk substitutes, their ingredients and nutritional values in 100 g of product as stated on the packaging of the selected products

| Brand | Name | Ingredients | Energy [kcal] | Protein  [g] | Carbohydrates [g] | Sugars [g] | Fat  [g] | Saturated fat [g] |
| --- | --- | --- | --- | --- | --- | --- | --- | --- |
| The good little Cook | Almond MLK | Water, 20% almonds | - | - | - | - | - | - |
| alpro | Almond original | Water, sugar, almond (2%), tri-calcium phosphate, sea salt, stabilisers (locust bean gum, gellan gum), emulsifier (sunflower lecithin), vitamins B2, B12, E, D2) | 24 | 0.5 | 3 | 3 | 1.1 | 0.1 |
| Provamel | Organic almond drink | Water, almond* (7%), sea salt | 33 | 0.9 | 0.2 | 0.2 | 3 | 0.3 |
| The good little Cook | Carob almond MLK | Water, almonds, carob, dates, maple syrup | - | - | - | - | - | - |
| Provamel | Organic cashew drink | Water, roasted cashew (6%), agave syrup (3.5%), sea salt | 47 | 0.9 | 4.4 | 2.9 | 2.8 | 0.5 |
| alpro | Coconut original | Water, coconut milk (5.3%) (coconut cream, water), rice (3.3%), tri-calcium phosphate, stabilisers (carrageenan, guar gum, Xanthan gum), sea salt, vitamins (B12, D2), flavourings | 20 | 0.1 | 2.7 | 1.9 | 0.9 | 0.9 |
| alpro | Hazelnut original | Water, sugar, hazelnuts (2.5%), tri-calcium phosphate, sea salt, stabilisers (locust bean gum, gellan gum), emulsifier (sunflower lecithin), vitamins B2, B12, E, D2) | 29 | 0.4 | 3.1 | 3.1 | 1.6 | 0.2 |
| Braham & Murray | Hemp Milk Unsweetened | Water, hemp cream (3%), tri-calcium phosphate, emulsifier (sucrose ester), natural flavouring, stabiliser (xanthan gum), sea salt, stabiliser (gellan gum), vitamin D2 | 23 | <0.1 | <0.1 | <0.1 | 2.7 | 0.3 |
| Provamel | Organic macadamia drink | Water, macadamia nuts (4%), agave syrup (3.5%), sea salt | 34 | 0.5 | 2.4 | 2.1 | 2.4 | 0.4 |
| Oatly | Organic oat drink | Oat base (water,oats 10%), sea salt | 35 | 1 | 6.5 | 4 | 0.5 | 0.1 |
| EcoMil | Quinoa drink | Water, quinoa(7%), agave syrup*, corn maltodextrin, almond oil | 46 | 1.5 | 3.7 | 2.5 | 2.8 | 0.7 |
| Vitariz | Organic rice drink natural | Organic rice, water, organic sunflower oil, sea salt | 64 | 0.7 | 10.5 | 9.5 | 1.9 | 0.2 |
| Rude Health | Organic brown rice drink | Water, organic brown rice (14%), sunflower oil, sea salt. | 59 | 0.3 | 11 | 5 | 1.3 | 0.4 |
| Provamel | Organic soya drink, calcium | Water, hulled soya beans (7,2%), apple concentrate* (3,3%), algue lithothamnium calcereum (0,4%), sea salt | 188 | 3.7 | 2.4 | 2.4 | 2.1 | 0.4 |
| Sojade | Plain UHT organic soya drink | Water, hulled organic soya beans (6.5%) | 154 | 3.8 | 0.45 | 0.45 | 2.1 | 0.3 |
| alpro | Soya organic, wholebean | Water, hulled soya beans (8%). | 132 | 3.3 | 2.4 | 2.4 | 1.9 | 0.3 |
| alpro | Soya original | Water, hulled soya beans (6%), sugar, acidity regulators (mono-potassium phosphate, di-potassium phosphate), calcium carbonate, flavouring, sea salt, stabiliser (gellan gum), vitamins (B2, B12, D2) | 167 | 3 | 2.8 | 2.7 | 1.8 | 0.3 |
| Clona Dairy Product Ltd. | Fresh milk, pasteurised & homogenised | Milk | 64 | 3.3 | 4.8 | 4.8 | 3.5 | 2.3 |
